# Dr. Berry’s Death

**Published:** 1889-11

**Authors:** 


### Dr. Berry's Death.

THE DENTISTS OF THE CITY TAKE APPROPRIATE ACTION.

The dentists of Cincinnati held a meeting at the Dental College to take into consideration the death of Archibald Berry, D.D.S. There were present Drs. J. G. Cameron, H. A. Smith, J. I. Taylor, Wm. Taft, C. M. Wright, James Leslie, H. T. Smith, H. L. Moore, Frank Hunter, M. L. Milliken, D. W. Roudebush, E. Ware, J. S. Cassiday, D. W. Clancey, W. D. Phillips, G. Mollyneaux, M. H. Fletcher, A. G. Rose, E. Binford, C. H. Martin, R. J. Poore, G. W. Smith. Dr. J. G. Cameron, President; Dr. M. H. Fletcher, Secretary.

On motion the chair appointed Drs. James Leslie, C. M. Wright and D. W. Clancey a committee to take into consideration the death of Dr. A. Berry, who reported as follows: That in the death of Dr. Archibald Berry, who died in Boston, Mass., October 3, during a visit to that city, in the seventy-ninth year of his age, the dental profession has lost one of its prominent members. Shortly after his graduation he removed to Raymond, Mississippi, and practiced a number of years in that State He removed to this city about the year 1862, and secured by his ability and skill, a good practice, which he retained until within a few years on account of failing health. From the beginning of his dental career he took a warm interest in dental education, and did much to advance the interests of the Ohio College of Dental Surgery in Cincinnati, it being his Alma Mater, he being one of four of its first graduating class. He was one of the founders of the Mississippi Valley Dental Association, being the first society of dentists meeting for mutual instruction and improvement in the history of the profession. He was also prominent in the Ohio State Dental Society, the Mad River Valley Dental Society, the Odontological Society of Cincinnati, and others, He was known as a thinker and expressed himself with clearness and force. He had his peculiarities of manner, and was a truthful, earnest, and genial man. The papers he read at meetings secured the attention of his hearers, instructing the profession, and taught a liberality of imparting dental knowledge, much needed during the earlier years of his practice. In his private life he took great interest in every thing he thought conducive to the welfare of men—religion, temperance, and hygiene —having been an active member of organizations that had these results in view. We will miss his counsels, as the inevitable that is coming to us all has come to him. We kindly tender to his bereaved widow and relatives our sympathy in this dispensation of God's providence.

"*Resolved*, That the dentists of Cincinnati and vicinity attend the funeral of deceased; and that a copy of these proceedings be sent to the family and to the press.

"Dr. James Leslie,
"Dr. C. M. Wright,
"Dr. D. W. Clancey,
"Committee."

Some interesting remarks were made by Drs. H. A. Smith, D. W. Roudebush, James Leslie, and C. M. Wright. On motion this report and resolution were adopted:

"Dr. H. M. H. Fletcher,
"Secretary."

